# Three-Dimensional Mixed Convection Flow of Viscoelastic Fluid with Thermal Radiation and Convective Conditions

**DOI:** 10.1371/journal.pone.0090038

**Published:** 2014-03-07

**Authors:** Tasawar Hayat, Muhammad Bilal Ashraf, Hamed H. Alsulami, Muhammad Shahab Alhuthali

**Affiliations:** 1 Department of Mathematics, Quaid-i-Azam University, Islamabad, Pakistan; 2 Nonlinear Analysis and Applied Mathematics (NAAM) Research Group, Faculty of Science, King Abdulaziz University, Jeddah, Saudi Arabia; National Institute of Genomic Medicine, Mexico

## Abstract

The objective of present research is to examine the thermal radiation effect in three-dimensional mixed convection flow of viscoelastic fluid. The boundary layer analysis has been discussed for flow by an exponentially stretching surface with convective conditions. The resulting partial differential equations are reduced into a system of nonlinear ordinary differential equations using appropriate transformations. The series solutions are developed through a modern technique known as the homotopy analysis method. The convergent expressions of velocity components and temperature are derived. The solutions obtained are dependent on seven sundry parameters including the viscoelastic parameter, mixed convection parameter, ratio parameter, temperature exponent, Prandtl number, Biot number and radiation parameter. A systematic study is performed to analyze the impacts of these influential parameters on the velocity and temperature, the skin friction coefficients and the local Nusselt number. It is observed that mixed convection parameter in momentum and thermal boundary layers has opposite role. Thermal boundary layer is found to decrease when ratio parameter, Prandtl number and temperature exponent are increased. Local Nusselt number is increasing function of viscoelastic parameter and Biot number. Radiation parameter on the Nusselt number has opposite effects when compared with viscoelastic parameter.

## Introduction

Analysis of non-Newtonian fluids is an active area of research for the last few years. Such fluids represent many industrially important fluids including certain oils, shampoos, paints, blood at low shear rate, cosmetic products, polymers, body fluids, colloidal fluids, suspension fluids, pasta, ice cream, ice, mud, dough floor etc. In many fields such as food industry, drilling operations and bioengineering, the fluids, either synthetic or natural, are mixtures of different stuffs such as water, particle, oils, red cells and other long chain molecules. Such combination imparts strong rheological properties to the resulting liquids. The dynamic viscosity in non-Newtonian materials varies non-linearly with the shear rate; elasticity is felt through elongational effects and time-dependent effects. The fluids in these situations have been treated as viscoelastic fluids. Further, all the non-Newtonian fluids in nature cannot be predicted by single constitutive equation. Hence all the contributors in the field are using different models of non-Newtonian fluids in their theoretical and experimental studies (see [1]-[11] and several refs. therein). The boundary layer flows of non-Newtonian fluids in the presence of heat transfer have special importance because of practical engineering applications such as food processing and oil recovery. Especially the stretching flows in this direction are prominent in polymer extrusion, glass fiber and paper production, plastic films, metal extrusion and many others. After the pioneering works of Sakiadis [12] and Crane [13], numerous works have been presented for two-dimensional boundary layer flow of viscous and non-Newtonian fluids over a surface subject to linear and power law stretching velocities (see some recent studies [14]-[21]). It has been noted by Gupta and Gupta [22] that stretching mechanism in all realistic situations is not linear. For instance the stretching is not linear in plastic and paper production industries. Besides these the flow and heat transfer by an exponentially stretching surface has been studied by Magyari and Keller [23]. In this attempt the two-dimensional flow of an incompressible viscous fluid is considered. The solutions of laminar boundary layer equations describing heat and flow in a quiescent fluid driven by an exponentially permeable stretching surface are numerically analyzed by Elbashbashy [24]. Al- Odat et al. [25] numerically discussed the thermal boundary layer on an exponentially stretching surface with an exponential temperature distribution. Here magnetohydrodynamic flow is addressed. Nadeem and Lee [26] presented the steady boundary layer flow of nanofluid over an exponential stretching surface. Sajid and Hayat [27] examined the thermal radiation effect in the boundary layer flow and heat transfer of a viscous fluid. The flow is caused by an exponentially stretching sheet. The thermal radiation effect in steady hydromagnetic mixed convection flow of viscous incompressible fluid past an exponentially stretching sheet is examined by El-Aziz and Nabil [28]. Pal [29] carried out an analysis to describe mixed convection heat transfer in the boundary layer flow on an exponentially stretching continuous surface with an exponential temperature. Here analysis is given in the presence of magnetic field, viscous dissipation and internal heat generation/absorption. Khan and Sanajayand [30] investigated the heat and mass transfer effects of viscoelastic boundary layer flow over an exponentially stretching sheet in presence of viscous dissipation and chemical reaction. Bhattacharyya [31] numerically investigated the heat transfer boundary layer flow over an exponentially shrinking sheet. Shooting method is implemented here. Recently, Mukhopadhyay et al. [32] dealt with the boundary layer flow and heat transfer of a non-Newtonian fluid over an exponentially stretching permeable surface. Mustafa et al. [33] studied the boundary layer flow of nanofluid over an exponentially stretching sheet with convective boundary conditions. Flow and heat transfer for three-dimensional viscous flow over an exponentially stretching surface is discussed by Liu et al. [34]. Bhattacharyya et al. [35] studied the effects of thermal radiation in the flow of micropolar fluid past a porous shrinking sheet with heat transfer. The transient free convection interaction with thermal radiation of an absorbing emitting fluid along moving vertical permeable plate is discussed by Makinde [36]. Hayat et al. [37] considered a two-dimensional mixed convection boundary layer MHD stagnation point flow through a porous medium bounded by a stretching vertical plate with thermal radiation.

Literature survey indicates that the published studies about three-dimensional flow by an exponentially stretching surface are still scarce. To our knowledge, there is only one recent study by Liu et al. [34] which describes the three-dimensional boundary layer flow of a viscous fluid over an exponentially stretching surface. Thus motivation of present research is to venture further in the regime of three-dimensional mixed convection flow of viscoelastic fluid over an exponentially stretching surface with thermal radiation. The surface possess the convective type heat condition. No doubt the thermal radiation effects are significant in many environmental and scientific developments, for instance, in aeronautics, fire research, heating and cooling of channels, etc. It is found that radiative transport is often comparable and hence associated with that of convective heat transfer in several real-world applications. Therefore it is of great worth to the researchers to study combined radiative and convective flow and heat transfer aspects. Moreover, the skin friction coefficients for three-dimensional viscoelastic fluid have been computed which has not yet been available in the literature. This paper is structured into the following fashion. Section two consists of mathematical formulation and definitions of physical quantities of interest. Convergent series solutions of the involved nonlinear systems are developed in section three. The solutions in this section are developed by homotopy analysis method (HAM) [38]-[45]. Section four comprises discussion with respect to seven pertinent parameters involved in the solutions of velocity components and temperature. Section five syntheses the main observations.

## Mathematical Modelling

**Figure 1:**
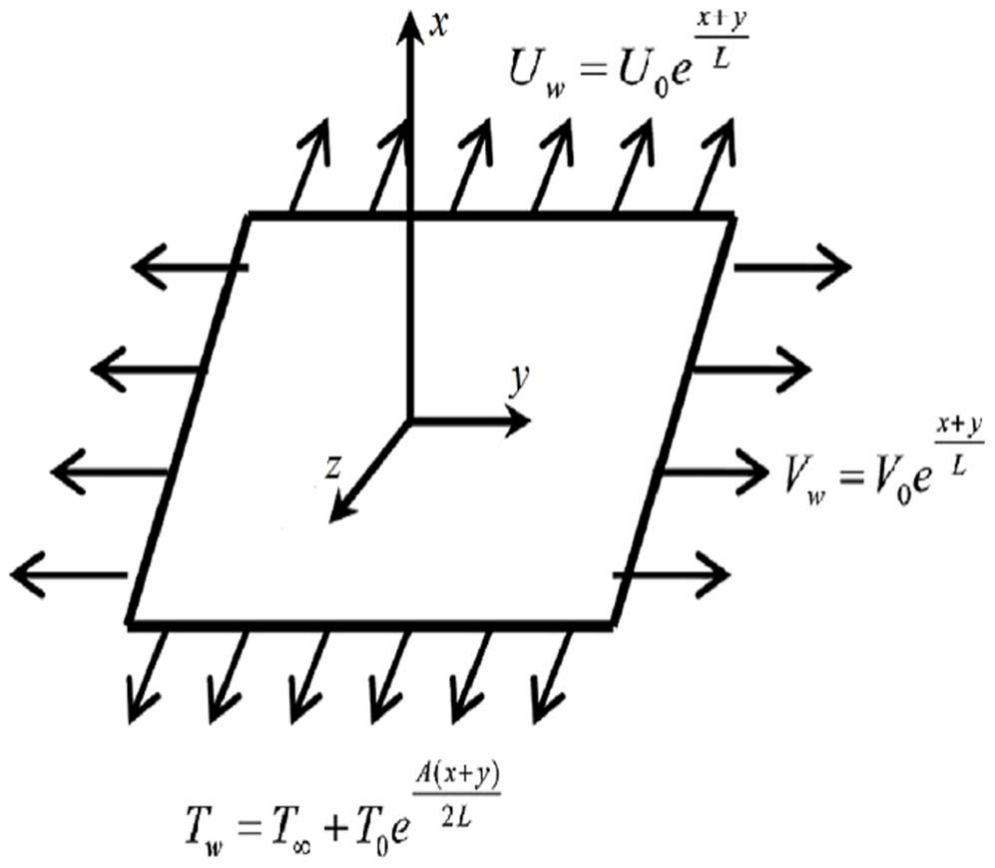
Geometry of Problem.

We consider three dimensional mixed convection boundary layer flow of second grade fluid passing an exponentially stretching surface. The surface coincides with the plane 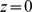 and the flow is confined in the region 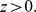 The surface also possess the convective boundary condition. Influence of thermal radiation through Rosseland's approximation is taken into account. Flow configuration is given below in Fig. 1.

The governing boundary layer equations for steady three-dimensional flow of viscoelastic fluid can be put into the forms (see Nazar and Latip [11]):
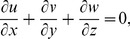
(1)

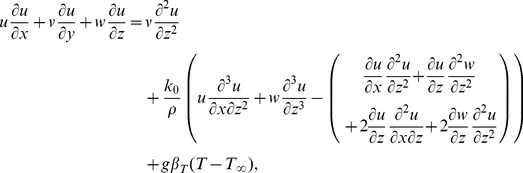
(2)

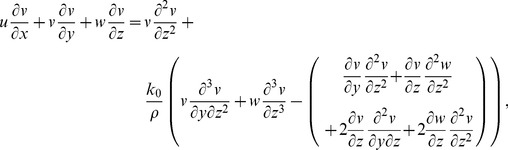
(3)

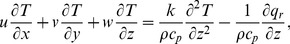
(4)


where 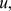 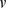 and 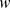 are the velocity components in the 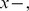 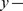 and 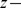 directions respectively, 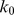 is the material fluid parameter, 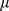 is the dynamic viscosity, 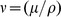 is the kinematic viscosity, 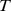 is the fluid temperature, 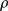 is the fluid density, 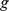 is the gravitational acceleration, 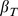 is thermal expansion coefficient of temperature, 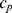 is the specific heat, 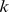 is the thermal conductivity and 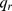 the radiative heat flux. Note that w-momentum equation vanishes by applying boundary layer assumptions (see Schlichting [46]).

By using the Rosseland approximation, the radiative heat flux 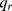 is given by 
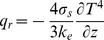
(5)


Where 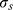
is the Stefan-Boltzmann constant and 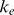 the mean absorption coefficient. By using the Rosseland approximation, the present analysis is limited to optically thick fluids. If the temperature differences are sufficiently small then Eq. (5) can be linearized by expanding 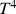 into the Taylor series about 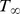, which after neglecting higher order terms takes the form:
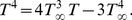
(6)


By using Eqs. (5) and (6), Eq. (4) reduces to
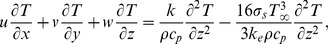
(7)


The boundary conditions can be expressed as 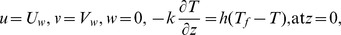


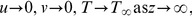
(8)


where subscript w corresponds to the wall condition, 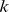
is the thermal conductivity, 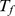 is the hot fluid temperature, 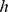
is the heat transfer coefficient and 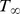
is the free stream temperature.

The velocities and temperature are taken in the following forms:
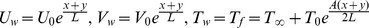
(9)


in which 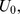 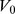 are the constants, 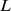 is the reference length and 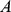
is the temperature exponent.

The mathematical analysis of the problem is simplified by using the transformations (Liu et al. [34]):
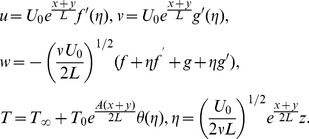
(10)


Incompressibility condition is now clearly satisfied whereas Eqs. (2)–(7) give
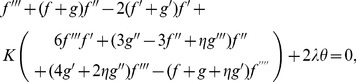
(11)

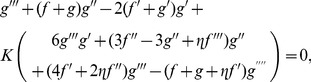
(12)

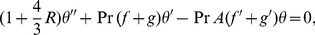
(13)

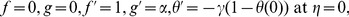
(14)

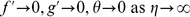
(15)


in which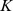 is the viscoelastic parameter, 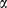 is the ratio parameter, 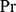
is the Prandtl number, 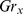
is the local Grashof number, 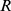
is the radiation parameter, 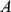
is the temperature exponent, 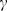 is the Biot number, 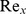 is the local Reynold number, 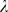 is the mixed convection parameter and prime denotes the differentiation with respect to 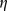. These can be defined as
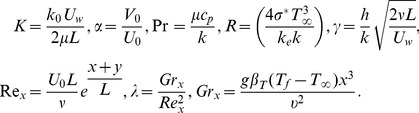
(16)


The skin-friction coefficients in the x and y directions are given by
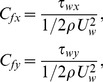
(17)


where
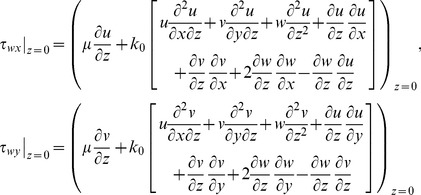
(18)


By using Eq. (18) in Eq. (17) the non-dimensional forms of skin friction coefficients are as follows:
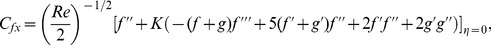
(19)

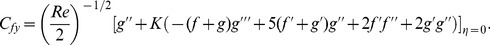
(20)


Further the local Nusselt number has the form 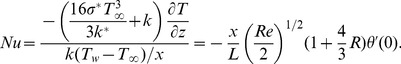
(21)


### Series Solutions

The initial guesses and auxiliary linear operators in the desired HAM solutions are 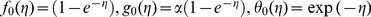
(22)

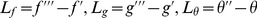
(23)


subject to the properties
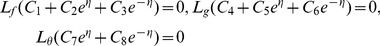
(24)


in which 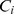 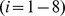 are the arbitrary constants, 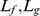 and 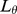 are the linear operators and 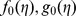 and 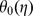 are the initial guesses.

Following the idea in ref. [38] the zeroth order deformation problems are 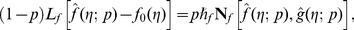
(25)

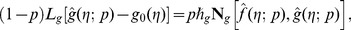
(26)

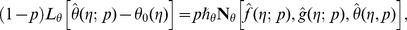
(27)

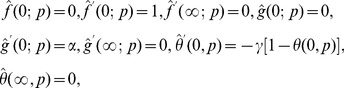
(28)


For 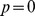 and 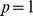 one has
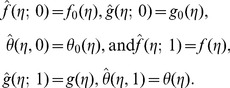
(29)


Note that when 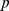 increases from 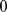 to 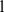 then 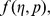 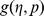 and 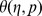 vary from 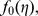 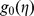 and 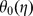 to 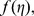 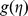
and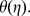 So as the embedding parameter 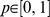 increases from 0 to 1, the solutions 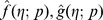 and 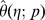 of the zeroth order deformation equations deform from the initial guesses 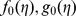 and 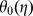
to the exact solutions
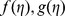
and 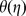
of the original nonlinear differential equations. Such kind of continuous variation is called deformation in topology and that is why the Eqs. (26-28) are called the zeroth order deformation equations. The values of the nonlinear operators are given below:
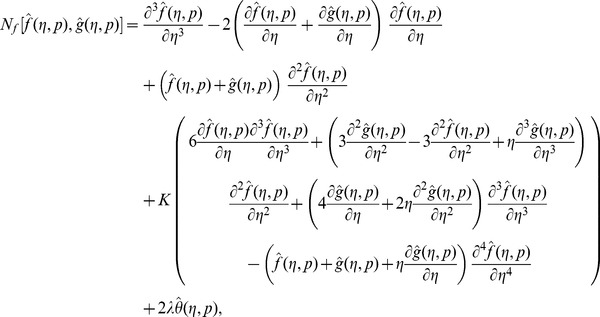
(30)

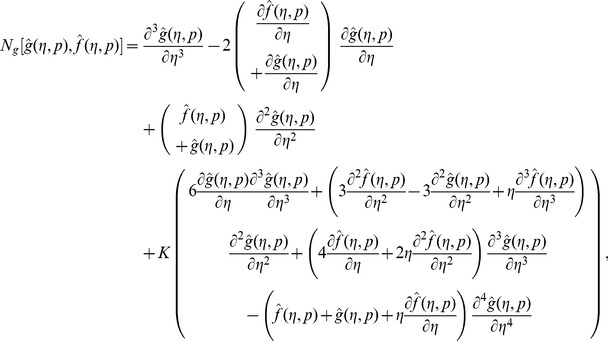
(31)

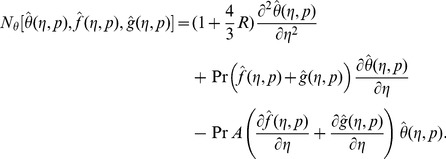
(32)


Here 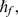 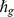
and 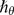 are the non-zero auxiliary parameters and 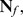 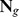
and 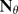 the nonlinear operators. Taylor series expansion gives
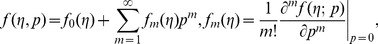
(33)

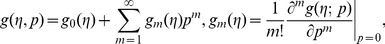
(34)

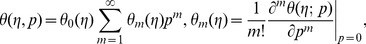
(35)


where the convergence of above series strongly depends upon 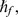 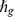
and 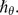 Considering that 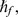 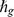 and 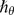
are chosen in such a manner that Eqs. (33)-(35) converge at 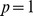 then
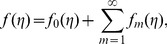
(36)

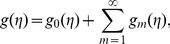
(37)

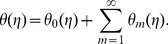
(38)


The corresponding problems at mth order deformations satisfy
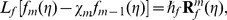
(39)

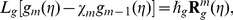
(40)

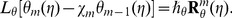
(41)

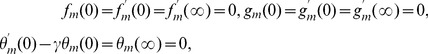
(42)

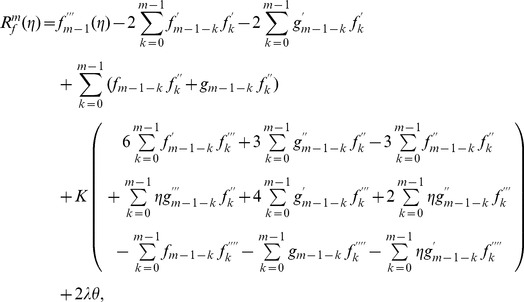
(43)

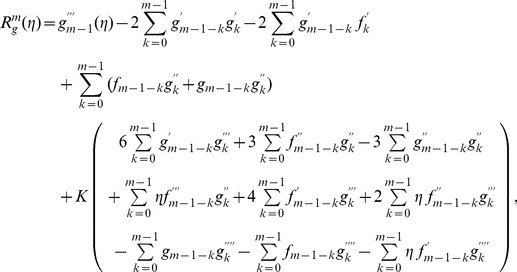
(44)

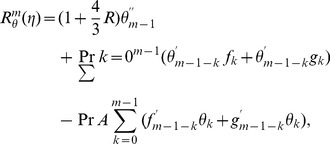
(45)


The mth order deformation problems have the solutions
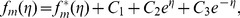
(46)

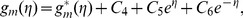
(47)

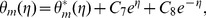
(48)


where the special solutions are 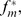 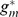 and 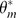.

## Convergence Analysis

**Figure 2:**
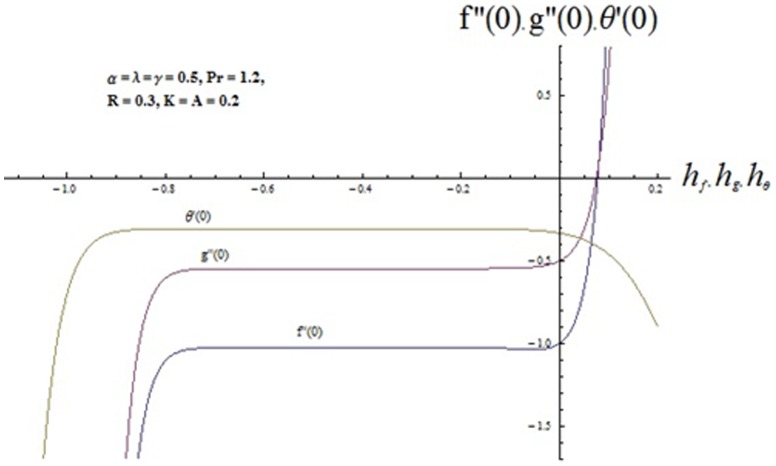
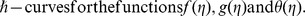

We recall that the series (36-38) contain the auxiliary parameters 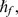 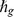 and 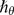. These parameters are useful to adjust and control the convergence of homotopic solutions. Hence the 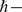 curves are sketched at 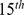 order of approximations in order to determine the suitable ranges for 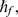 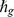 and 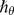. Fig. 2 denotes that the range of admissible values of 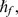 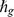 and 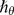 are 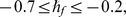 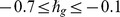 and 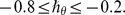 Table 1 shows that the series solutions converge in the whole region of 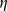 when 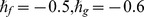 and 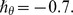


**Table 1:** Convergence of series solutions for different order of approximations when 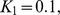 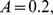 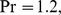 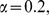 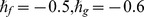 and 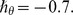

Order of aproximations	1	5	10	15	20	25
-f′′(0)	1.06111	1.02482	1.02609	1.02623	1.02618	1.02618
-g′′ (0)	0.544444	0.548057	0.548092	0.548043	0.548053	0.548053
-θ′ (0)	0.317778	0.305581	0.305729	0.305744	0.305738	0.305738

## Discussion of Results

**Figure 3:**
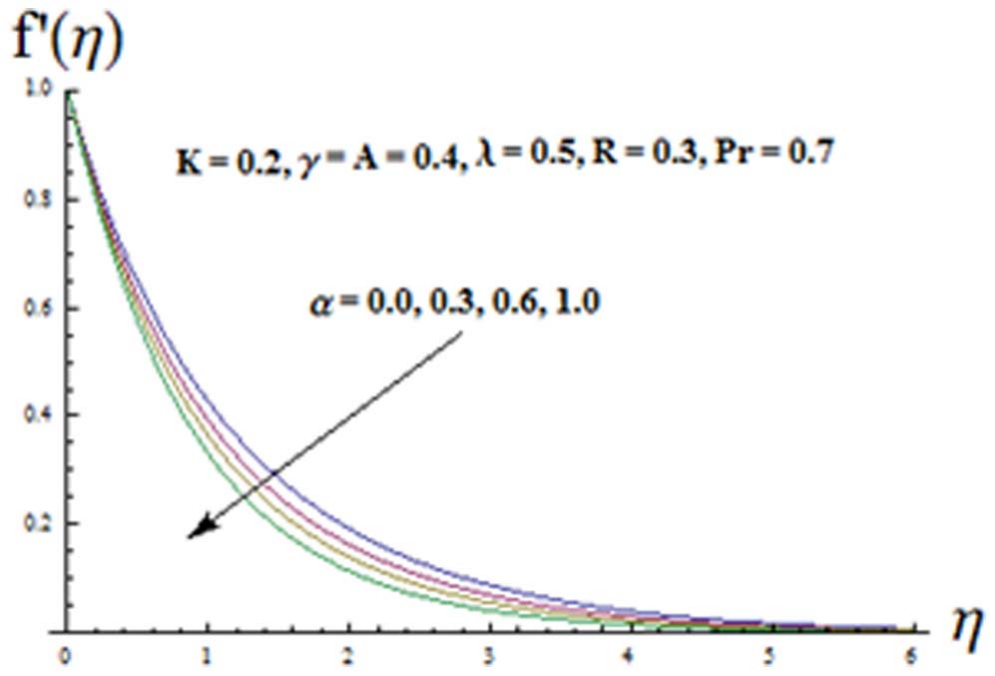
Influence of 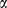 on the velocity 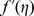.

**Figure 4:**
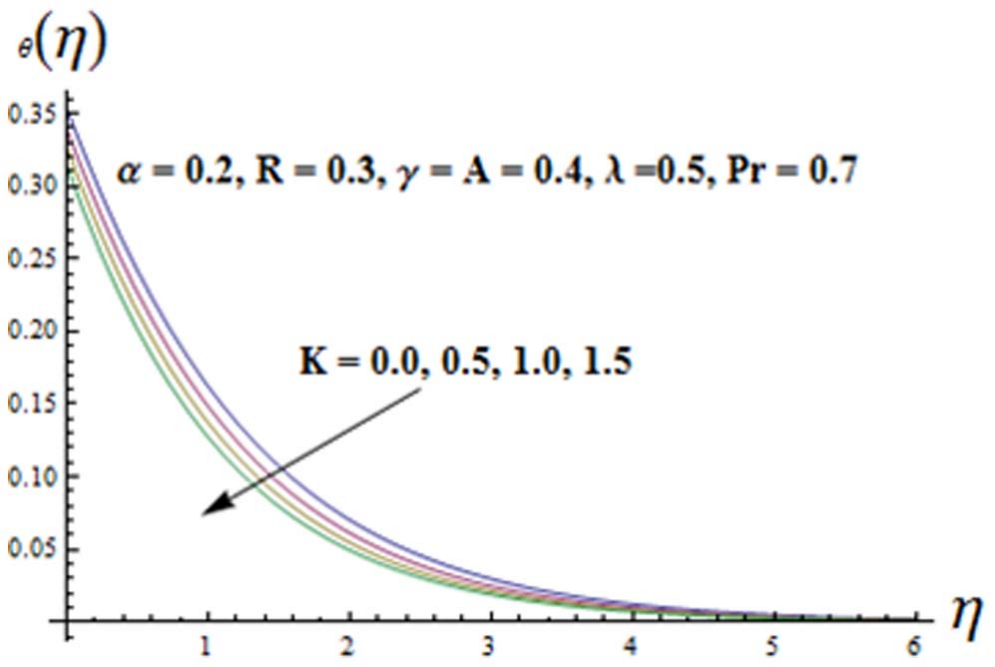
Influence of K on the velocity 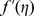.

**Figure 5:**
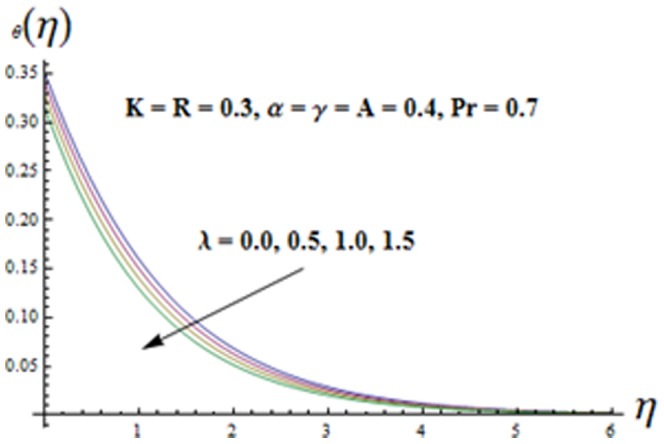
Influence of 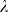 on the velocity 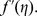.

**Figure 6:**
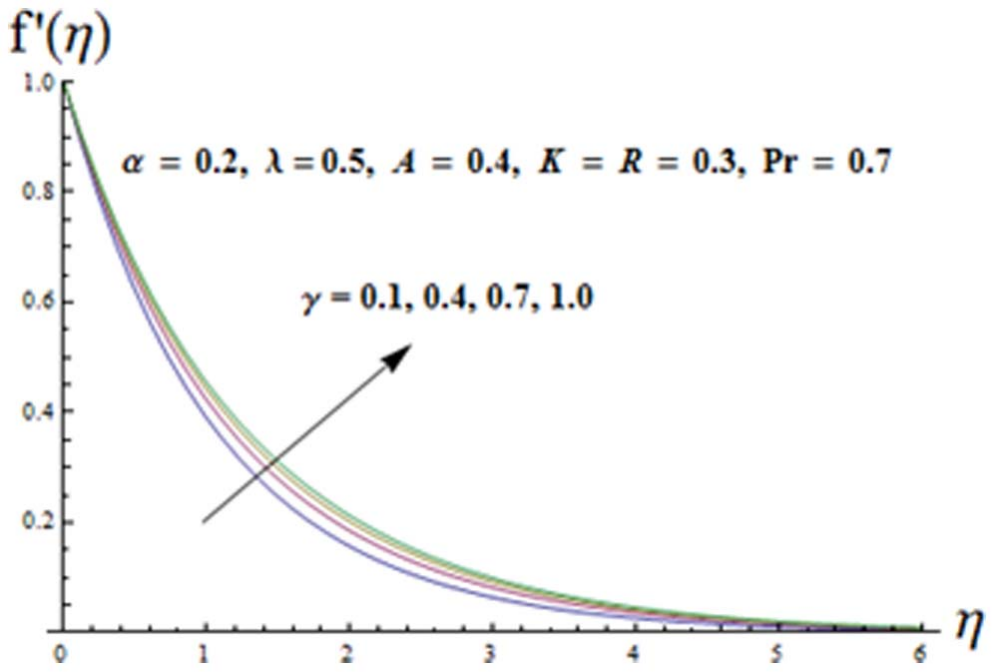
Influence of 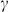 on the velocity 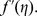.

**Figure 7:**
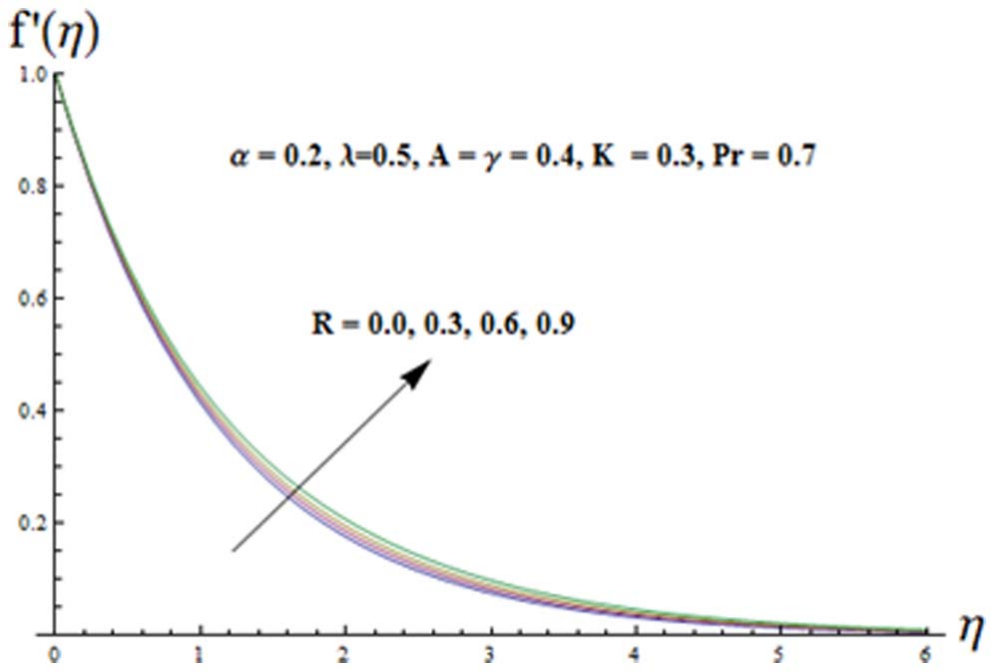
Influence of R on the velocity 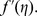.

**Figure 8:**
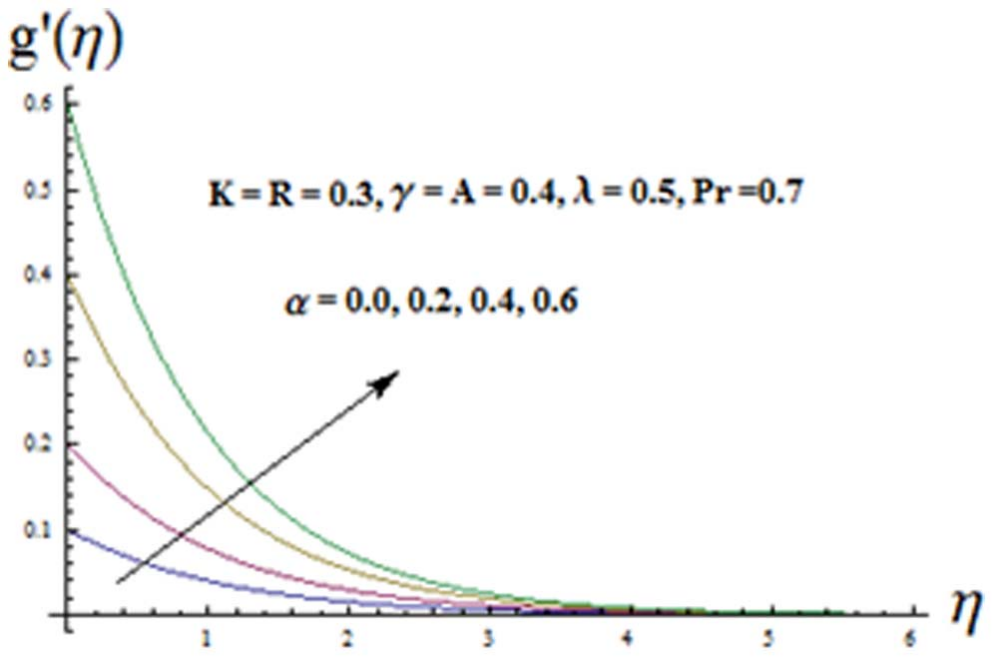
Influence of 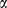 on the velocity 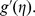.

**Figure 9:**
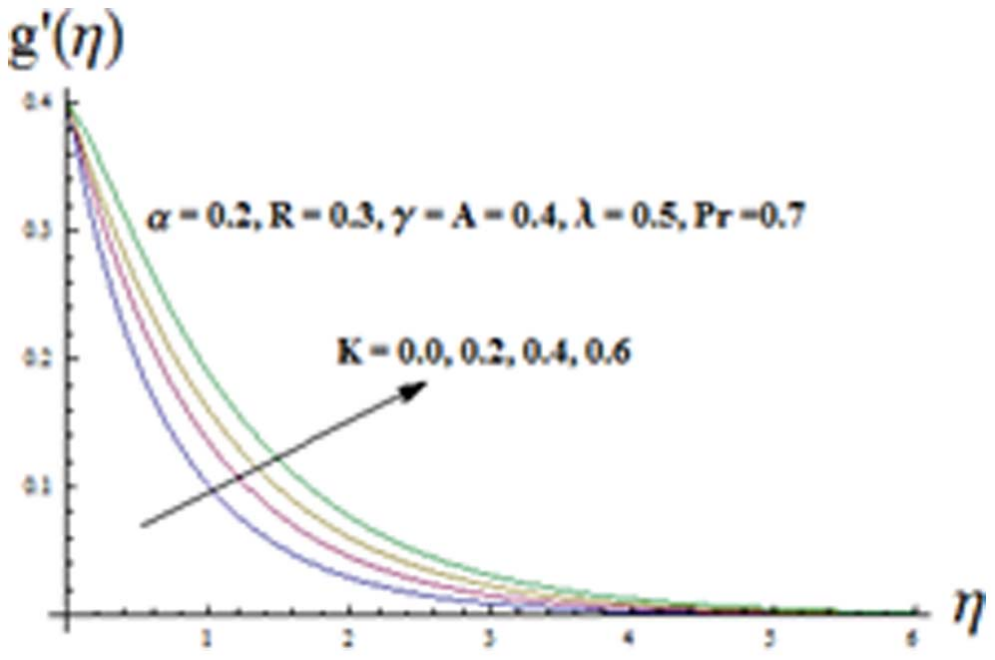
Influence of K on the velocity 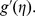.

**Figure 10:**
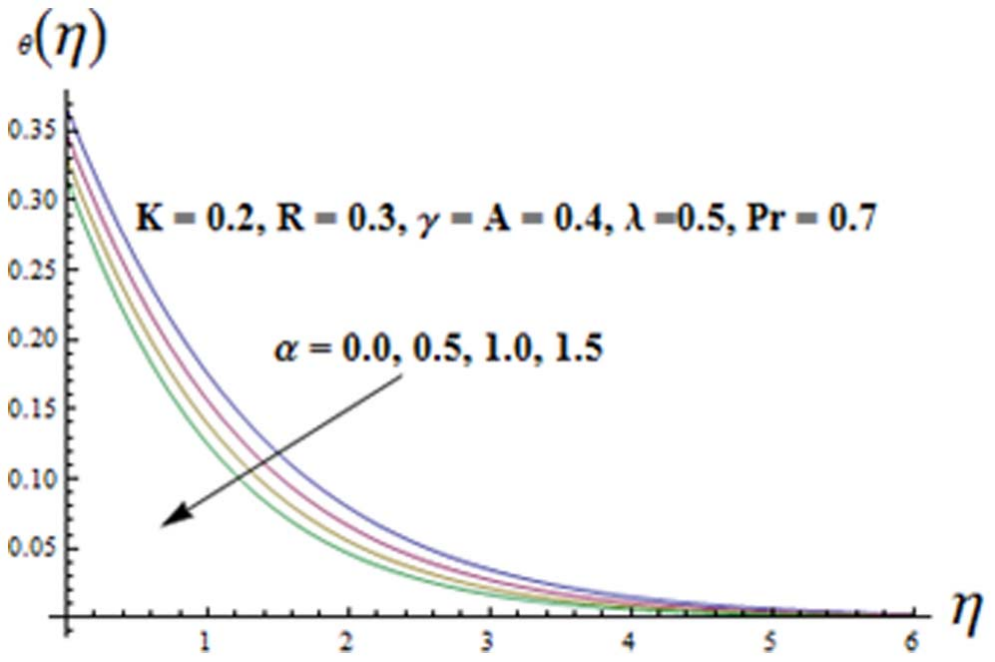
Influence of 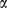 on the temperature 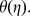.

**Figure 11:**
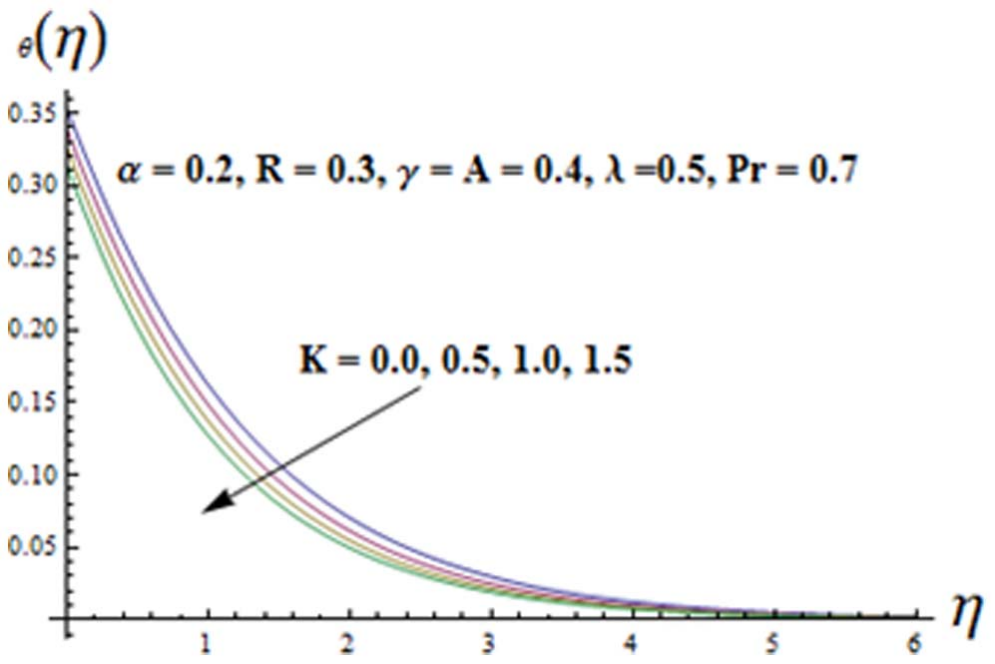
Influence of K on the temperature 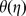.

**Figure 12:**
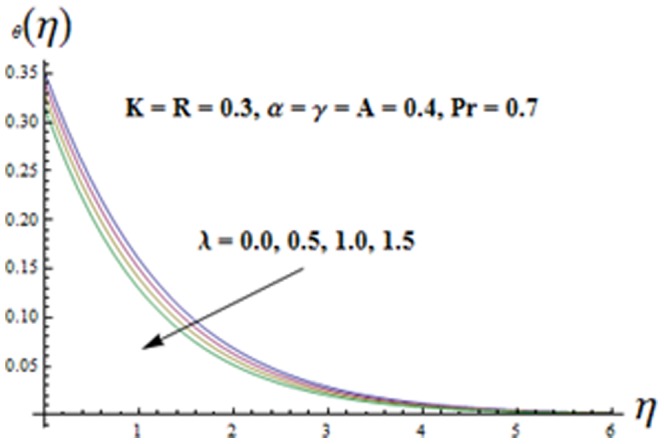
Influence of 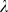 on the temperature 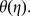.

**Figure 13:**
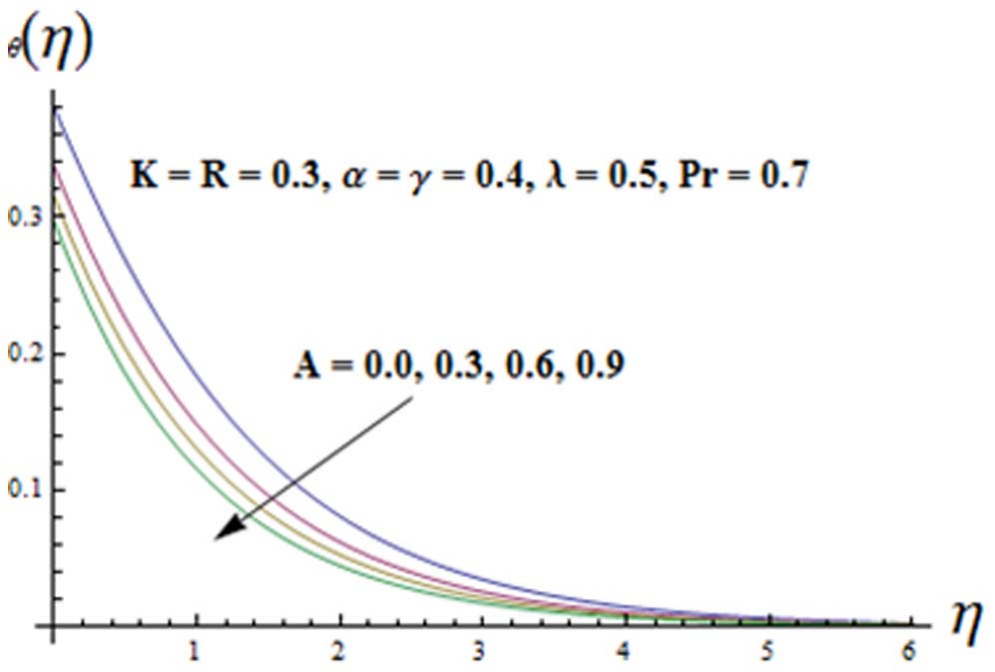
Influence of 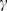 on the temperature 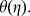.

**Figure 14:**
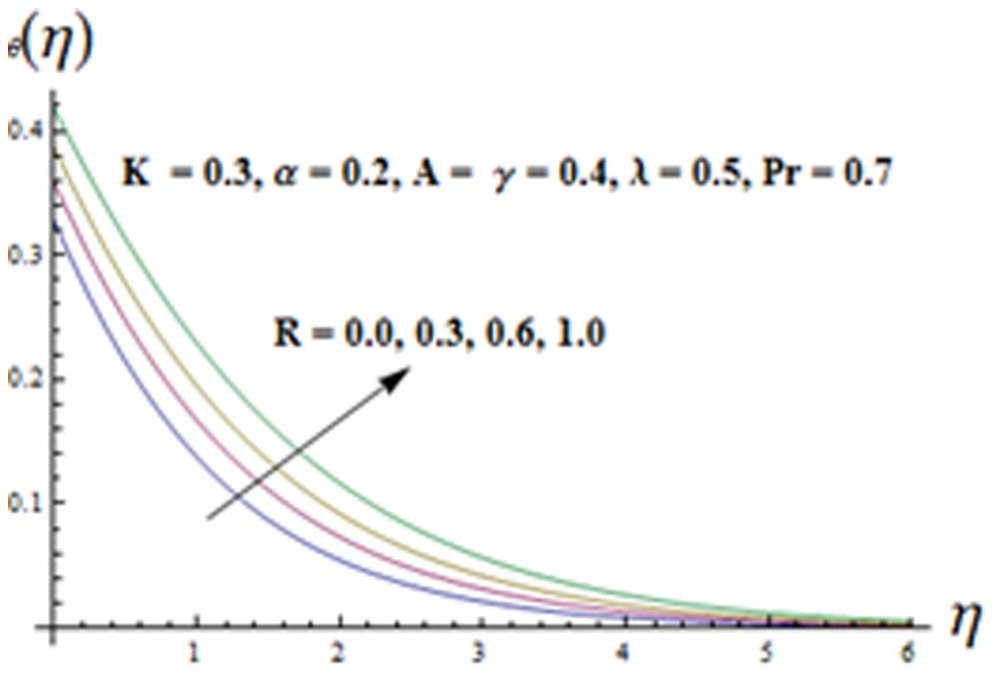
Influence of A on the temperature 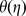.

**Figure 15:**
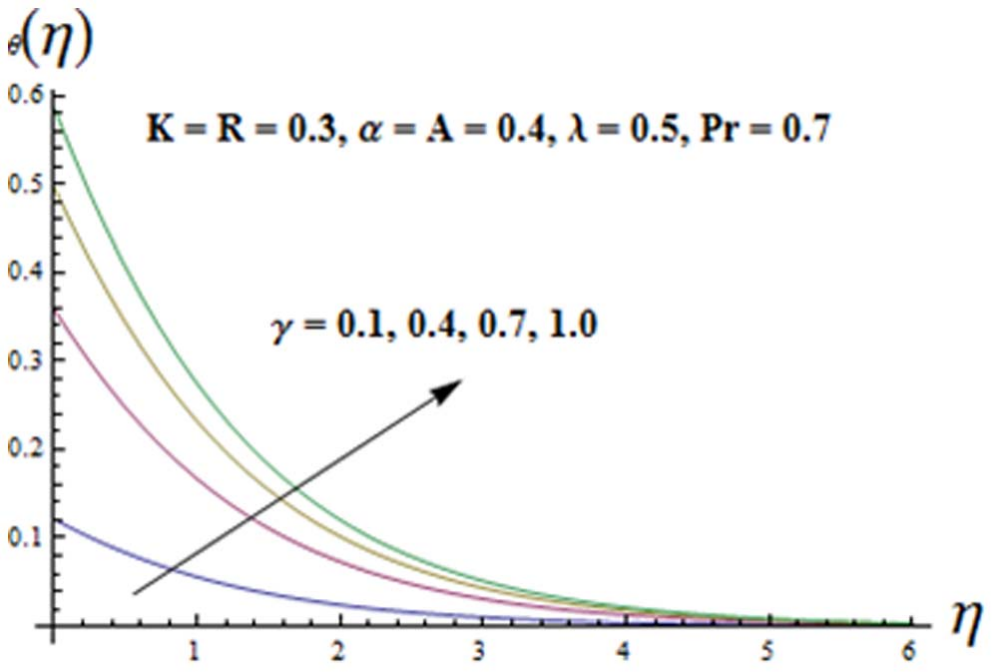
Influence of R on the temperature 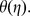.

**Figure 16:**
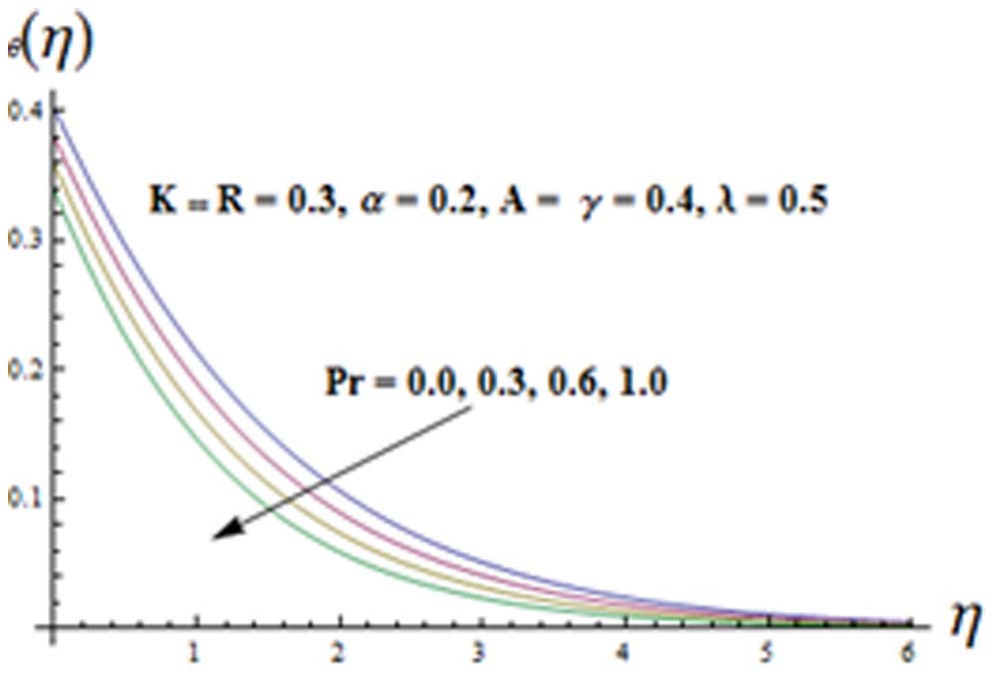
Influence of 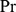 on the temperature 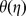.

The effects of ratio parameter 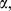 viscoelastic parameter 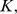 mixed convection parameter 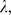 Biot number 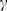 and radiation parameter 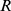 on the velocity component 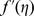 are shown in the Figs. 3-7. It is observed from Fig. 3 that velocity component 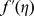 and thermal boundary layer thickness are decreasing functions of ratio parameter 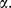 This is due to the fact that with the increase of ratio parameter 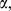 the x-component of velocity coefficient decreases which leads to a decrease in both the momentum boundary layer and velocity component 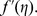 Fig. 4 illustrates the influence of viscoelastic parameter 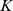 on the velocity component 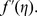 It is clear that both the boundary layer and velocity component 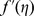 increase when the viscoelastic parameter increases. Influence of mixed convection parameter 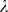 on the velocity component 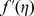 is analyzed in Fig. 5. Increase in mixed convection parameter 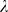 shows an increase in velocity component 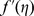. This is due to the fact that the buoyancy forces are much more effective rather than the viscous forces. Effects of Biot number 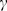 and the radiation parameter 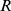 on the velocity component 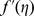 can be predicted from Figs. 6 and 7. These Figs. depict that the influences of 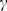 and 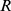 on both the velocity component 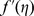 and thermal boundary layer thickness are similar i.e. there is increase in these quantities. Figs. 8 and 9 illustrate the variations of ratio parameter 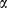 and viscoelastic parameter 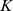 on the velocity component 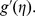 Variation of ratio parameter 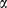
is analyzed in Fig. 8. Through comparative study with Fig. 3 it is noted that 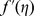 decreases while 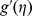 increases when 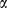 increases. Physically, when 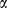 increases from zero, the lateral surface starts moving in y-direction and thus the velocity component 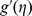 increases and the velocity component 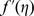 decreases. Fig. 9 is plotted to see the variation of viscoelastic parameter 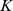 on the velocity component 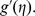 It is found that both the velocity component 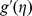 and momentum boundary layer thicknesses are increasing functions of 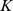. It is revealed from Figs. 4 and 9 that the effect of 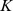 on both the velocities are qualitatively similar. Figs. 10-16 are sketched to see the effects of ratio parameter 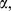 viscoelastic parameter 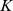, the temperature exponent 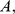 Biot number 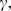 mixed convection parameter 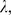 Radiation parameter and Prandtl number 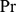 on the temperature 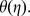 Fig. 10 is drawn to see the impact of ratio parameter 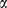 on the temperature 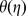. It is noted that the temperature 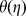 and also the thermal boundary layer thickness decrease with increasing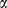. Variation of the viscoelastic parameter 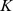 on the temperature 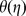 is shown in Fig. 11. Here both the temperature and thermal boundary layer thickness are decreasing functions of 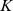. Variation of mixed convection parameter 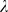 is analyzed in Fig.12. It is seen that both the temperature 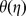 and thermal boundary layer thickness are decreasing functions of mixed convection parameter 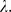 Fig.13 presents the plots for the variation of Biot number 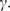 Note that 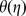 increases when 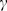 increases. The thermal boundary layer thickness is also increasing function of 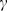. It is also noted that the fluid temperature is zero when the Biot number vanishes. Influence of temperature exponent 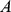 is displayed in Fig. 14. It is found that both the temperature 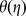 and thermal boundary layer thickness decrease when A is increased. Also both the temperature 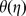 and thermal boundary layer thickness are increasing functions of thermal radiation parameter 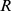 (see Fig. 15). It is observed that an increase in 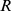 has the ability to increase the thermal boundary layer. It is due to the fact that when the thermal radiation parameter increases, the mean absorption coefficient 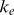 will be decreased which in turn increases the divergence of the radiative heat flux. Hence the rate of radiative heat transfer to the fluid is increased and consequently the fluid temperature increases. Fig. 16 is plotted to see the effects of 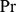 on 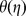. It is noticed that both the temperature profile and thermal boundary layer thickness are decreasing functions of 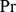. In fact when 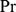 increases then thermal diffusivity decreases. This indicates reduction in energy transfer ability and ultimate it results in the decrease of thermal boundary layer.

Table 1 presents the numerical values of 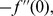 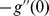 and 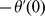 for different order of approximations when 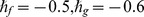 and 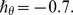 It is seen that the values of 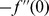 and 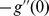 converge from 20th order of deformations whereas the values of 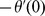 converge from 25th order approximations. Further, it is observed that we have to compute less deformations for the velocities in comparison to temperature for convergent series solutions. Table 2 includes the values for comparison of existing solutions with the previous available solutions in a limiting case when 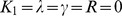 and 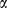 varies. This Table presents an excellent agreement with the previous available solutions. Table 3 is computed to see the influences of viscoelastic parameter 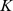 and ratio parameter 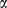 on skin friction coefficients in the x and y directions. It is noted that 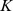 has quite opposite effect on skin friction coefficients while quite similar effect is seen within the increase of ratio parameter 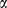. Table 4 examines the impact of viscoelastic parameter 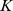, mixed convection parameter 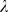, ratio parameter 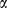, Biot number 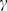, radiation parameter 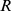, Prandtl number 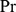 and temperature exponent 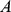 on the local Nusselt number (rate of heat transfer at the wall). It is noted that the value of rate of heat transfer increases for larger viscoelastic parameter 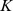, mixed convection parameter 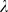, ratio parameter 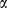, Biot number 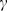, Prandtl number 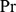 and temperature exponent 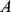 while it decreases through an increase in radiation parameter R.

**Table:** **Table 2.** Comparative values of 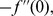 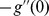 and 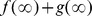 for different values 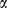 when 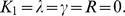

Liu et al. [34]				Present results		
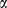	-f′′′ (0)	-g′′ (0)	f(∞)+g(∞)	-f′′ (0)	-g′′ (0)	f(∞)+g(∞)
0.0	1.28180856	0	0.90564383	1.28181	0	0.90564
0.50	1.56988846	0.78494423	1.10918263	1.56989	0.78494	1.10918
1.00	1.81275105	1.81275105	1.28077378	1.81275	1.81275	1.28077

**Table 3:** Values of skin friction coefficients for different values of K and α when λ = γ = 0.5, R = 0.3, Pr = 1.2 and A = 0.2.

K	α	- 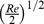 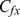	- 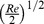 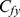
0.0	0.5	4.95289	4.37363
0.2		5.16586	3.97055
0.3		5.42622	3.96130
0.3	0.0	3.72170	1.65409
	0.2	4.30247	2.34617
	0.5	5.42622	3.96130

Table 2. Comparative values of 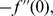 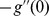 and 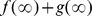 for different values 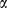 when 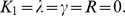


**Table 4:** Values of local Nusselt number 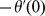 for different values of the parameters 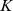, 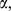 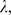 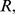 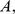 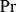 and 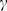.

K	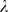	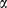	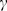	R	Pr	A	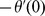
0.0	0.5	0.5	0.5	0.3	1.2	0.2	0.297492
0.3							0.308234
0.5							0.311853
0.2	0.0						0.303062
	0.3						0.304775
	0.5						0.305738
0.2	0.5	0.0					0.282007
		0.3					0.297135
		0.5					0.305738
			0.1				0.0885730
			0.3				0.216850
			0.5				0.305738
0.2	0.5	0.5	0.5	0.0			0.329701
				0.3			0.305738
				0.5			0.292750
0.2	0.5	0.5	0.5	0.3	1.0		0.292152
					1.2		0.305738
					1.5		0.321826
0.2	0.5	0.5	0.5	0.3	1.2	0.0	0.288530
						0.2	0.305738
						0.5	0.325492

## Conclusions

Three-dimensional mixed convection flow of viscoelastic fluid over an exponentially stretching surface is analyzed in this study. The analysis is carried out in the presence of thermal radiation subject to convective boundary conditions. The main observations can be summarized as follows:

- Influence of ratio parameter 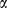 on the velocities 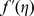 and 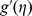 is quite opposite. However the effect of viscoelastic parameter 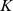 on the velocities 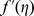 and 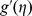 is qualitatively similar.
- Momentum boundary layer thickness increases for 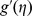 when ratio parameter 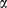 is large. Effect of 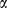 on 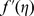 is opposite to that of 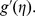

- Velocity component 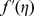 is increasing function of mixed convection parameter 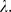 However 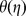 decreases with an increase of mixed convection parameter 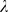. The impact of Biot number 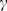 and radiation parameter 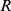 on 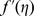 and 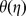 are qualitatively similar.
- Momentum boundary layer is an increasing function of mixed convection parameter 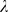 while thermal boundary layer is decreasing function of mixed convection parameter 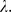

- Increase in Prandtl number decreases the temperature 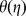.
- Thermal boundary layer thickness decreases when ratio parameter 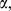 viscoelastic parameter 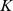, mixed convection parameter 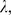 Prandtl number 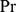 and temperature exponent 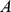 are increased.
- Influence of viscoelastic parameter 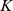 on the x and y direction of skin friction coefficients is opposite.
- Both components of skin friction coefficient increase through an increase in ratio parameter 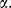

- Local Nusselt number is an increasing function of Prandtl number 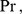 ratio parameter 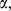 viscoelastic parameter 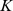, mixed convection parameter 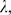 Biot number 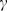 and temperature exponent 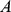 while it decreases for radiation parameter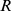.

## Supporting Information

File S1 Appendix.
(DOCX)
