# Supplementary material for: Three-Dimensional Mixed Convection Flow of Viscoelastic Fluid with Thermal Radiation and Convective Conditions
Source: PLoS One. 2014 Mar 7;9(3):e90038. doi: 10.1371/journal.pone.0090038 (PMC3946481; doi:10.1371/journal.pone.0090038)
Supplement: File S1 — Appendix. (DOCX) [file pone.0090038.s001.docx]

Appendix

Here we present briefly the involved steps in the derivation of governing equations.


Using the above equations and dimensionless variables we get Eq. (11).
